# Beyond Capacity: Resilience of Intensive Care Staff During the COVID‐19‐Related Near‐Disaster in Sweden

**DOI:** 10.1111/nicc.70251

**Published:** 2025-11-17

**Authors:** Per Kolton Nyberg, Mattias Günther, Ami Fagerdahl, Andreas Älgå

**Affiliations:** ^1^ Department of Anesthesia and Intensive Care Södersjukhuset Stockholm Sweden; ^2^ Department of Clinical Science and Education Södersjukhuset, Karolinska Institutet Stockholm Sweden; ^3^ Wound Centre, Södersjukhuset Stockholm Sweden; ^4^ Department of Surgery Södersjukhuset Stockholm Sweden; ^5^ Department of Global Public Health Karolinska Institutet Stockholm Sweden

**Keywords:** disaster, intensive care unit, moral distress, organisational adaptation

## Abstract

**Background:**

Intensive care units (ICUs) often operate near full capacity, limiting flexibility during crises. As geopolitical instability increases in Europe, healthcare systems must be prepared to expand and adapt under extreme pressure.

**Aim:**

To explore how ICU staff in Sweden—a country with traditionally low disaster preparedness—managed a near‐disaster situation and identify lessons for future crises.

**Study Design:**

A qualitative descriptive study using semi‐structured interviews and inductive content analysis. Eleven ICU staff members (anaesthesiologists, ICU nurses and nurse assistants) from three Stockholm hospitals were interviewed about their experiences during the 2020 COVID‐19 surge.

**Results:**

Three main domains emerged: organisational, team and individual levels. Participants described chaotic ICU expansions, limited supplies and unfamiliar equipment. Inconsistent staff continuity and lack of onboarding for redeployed personnel created stress and hindered care. Moral distress was widespread, fuelled by an overwhelming workload and poor recovery conditions. A voluntary emergency contract helped maintain staffing levels and morale. ICU staff took on leadership roles, educated peers and created ad hoc systems to manage extreme conditions—sometimes while wearing military‐grade gas masks. While peer support strengthened team cohesion, feelings of isolation and professional fragmentation also emerged.

**Conclusions:**

Near‐disaster conditions require visible local leadership, clear communication and a governance model that integrates frontline expertise. Rapid onboarding, scalable mental health support and clearly defined disaster activation protocols are key to system resilience.

**Relevance to Clinical Practice:**

ICU staff must be equipped to lead in crisis. Empowering experienced personnel to guide redeployed teams, ensuring rest and recovery opportunities and supporting staff psychologically can mitigate long‐term harm. This study provides practical insights for near‐disaster preparedness in high‐income, low‐readiness settings and supports the development of ICU protocols that balance hierarchical leadership with clinical agility.

**Impact Statements**

- What is known about the topic
  ○ Intensive care units (ICUs) often operate near capacity, leaving little room to scale up during crises.
  ○ Disaster preparedness research in high‐income countries tends to focus on protocols and infrastructure, not lived staff experience.
  ○ Sweden has historically low levels of disaster readiness and limited recent experience with large‐scale health crises.
- What this paper adds
  ○ Provides a first in‐depth qualitative account of Swedish ICU staff managing a near‐disaster without formal disaster activation.
  ○ Identifies how ICU teams assumed leadership, trained others and maintained morale despite fragmented governance and resource scarcity.
  ○ Offers actionable recommendations for hybrid governance, rapid onboarding and psychological support to improve resilience in future crises.

## 1 Introduction

The intensive care unit (ICU) is a high‐resource environment caring for the most critically ill patients that often run at near or full capacity during times of regular activity. When patient surges from a disaster occur, the ICU is at risk of not having the functional reserve to provide the usual standard of care. This may necessitate adjusting the model of care from a usual standard of care to a crisis standards of care model in order to treat a larger number of patients, often with limited resources [1]. The defence healthcare system of European high‐income countries like Sweden strongly depends on the support of civilian healthcare during increased levels of mobilisation [2]. During the cold war era, Sweden was able to mobilise all available resources to defend the country. However, it has lately been indicated that Swedish surgical mass casualty incident preparedness has decreased substantially since the cessation of the cold war.

## 2 Background/Justification for Study

A nationwide survey showed that the mass casualty incident preparedness of Swedish emergency care hospitals needed further attention and that a national strategy for trauma care in disaster management was necessary [2]. Most hospitals train their trauma teams with exercises including a limited number of simulated casualties, but few hospitals train for mass casualty incidents, focusing on the hospital's entire ability to deal with a very large number of trauma casualties. A question that has not been addressed is the endurance of Swedish hospitals to sustain in disaster mode [2]. This ability is relevant considering the ongoing war in Ukraine, where it has been shown that the ICU contingent changed significantly: the vast number of trauma patients was distributed to hospitals which had never managed trauma before, requiring education of personnel and several adaptations [3].

Effectively evaluating preparedness before a crisis occurs is essential to achieve optimal resilience. Surveys focusing on formal attributes have identified strengths and weaknesses in the Swedish system [4]. However, resilience in disaster settings is not only about physical resources and formal structures—it also encompasses the capacity of healthcare staff and organisations to adapt, recover, and maintain function when faced with extraordinary pressure [1, 5]. In the ICU context, resilience can be understood as the interplay between systemic preparedness and the individual healthcare worker's ability to adapt to high workloads, resource shortages and moral challenges. To date, resilience in Swedish ICUs has not been examined from the perspective of frontline staff. In Sweden, the official term is *disaster mode* (*katastrofläge*), denoting the highest level of hospital preparedness, whereas in much of the international literature the term *major incident* is commonly used to describe a comparable situation where healthcare demand significantly exceeds available resources.

Literature to date has predominately been based on table‐top disaster event exercises and theoretical planning approaches to disaster surge planning, such as staffing and resource shortages [5]. The point at which functional loss in the ICU is determined is relative to the ‘perceived normal’, which may vary between countries [1]. For this reason, we conducted a qualitative study on the perceptions of ICU staff in Stockholm, who were exposed to a near‐disaster in 2020. We define a near‐disaster as a critical care situation in which patient load, resource strain and operational disruption approached—but did not formally meet—the thresholds for activating official disaster protocols, thereby placing extreme demands on staff and infrastructure without systemic relief.

### 2.1 Aims and Objectives/Research Questions/Hypotheses

To understand how the ICU staff navigated the surge in patient load, in a country with traditionally low disaster preparedness.

### 2.2 Design and Methods

Written consent was obtained from all participants prior to the study. The recordings and transcripts were securely stored and only accessible to the research team. The investigation adheres to the principles outlined in the Declaration of Helsinki [6], and is reported according to the COREQ (COnsolidated criteria for REporting Qualitative research) checklist (Appendix S1).

### 2.3 Setting and Sample

Sweden is a European high‐income country with 21 regions. A region, in the context of Swedish municipal law, is a self‐governing unit with a geographical area of responsibility that corresponds to a county and with primary responsibility for providing public health care. Regions were previously referred to as ‘county councils’. In the form of government, they are referred to as ‘municipalities at the regional level’. This study involved hospitals in the Stockholm region. The Stockholm region has seven public hospitals, with a catchment area of 2 400 000 people [7]. The study was conducted in three ICUs in public emergency hospitals in the Stockholm region. Under normal conditions, these hospitals each have 6–10 ICU beds and a catchment area of 170 000–750 000 people [8, 9, 10]. All three hospitals provide emergency services, including internal medicine, surgery, orthopaedics, gynaecology and obstetrics. The so‐called emergency contract is described below. In exceptional situations, this agreement can be activated to temporarily override the Working Hours Act, allowing extended shifts with additional financial compensation for staff [11].

Participants were recruited using a stratified purposive sampling strategy to capture variation in perspectives across the ICU workforce [12]. Stratification factors included profession (anaesthesiologists, ICU nurses and assistant nurses), sex, age and years of ICU experience. This ensured representation of both junior and senior staff, as well as a balance between medical and nursing perspectives. Twelve individuals meeting these criteria were invited via email, of whom 11 consented to participate (Table 1). This stratified approach was chosen to enhance the transferability of findings by including a broad range of professional and demographic backgrounds relevant to ICU resilience.

**TABLE 1:** Characteristics of the participants (*n* = 11).

Age, median (range)	51 (31–62)
Work experience, median (range)	24 (10–40)
Years in ICU, median (range)	14 (4–39)
Male, *n* (%)	5 (45)
Female, *n* (%)	6 (55)
Anaesthesiologists, *n* (%)	3 (27)
ICU nurses, *n* (%)	5 (46)
ICU assistant nurses, *n* (%)	3 (27)

*Note:* This table presents demographic and professional background data of the ICU staff interviewed, including age, years of experience, sex distribution and professional roles (anaesthesiologists, ICU nurses and ICU assistant nurses).

Abbreviation: ICU, intensive care unit.

The study intended to investigate experiences for Swedish health care staff in the ICU during a real crisis. Sweden's last war was the Swedish–Norwegian war which ended in 1814, and Sweden has not experienced a real health crisis in modern times, apart from the COVID‐19 pandemic in which the number of ICU beds in Stockholm increased from about 100 to more than 250 [13]. We aimed to investigate the experiences gained in the ICU during the first wave of the COVID‐19 pandemic in Stockholm in 2020, with the intention of assessing factors needed to improve resilience in future disasters, and not pandemic‐specific factors, which are well described elsewhere.

During the first wave of the pandemic (March–April 2020), there was a global shortage of protective breathing masks. For this reason, the Swedish defence decided to distribute its standard protective mask (Protective Mask 90), designed for combat settings, among healthcare staff in the ICU. Protective Mask 90 protects the face and respiratory tract from biological and chemical warfare agents, radioactive dust, tear gas, soot particles and heat radiation. The filter provides at least 100 h of protection against chemical warfare agents. The mask, which weighs approximately 700 g, has both a drinking function and speech amplification.

### 2.4 Data Collection Tools and Methods

Semi‐structured interviews were conducted between December 2022 and October 2023 by the first author (PKN), a resident doctor in anaesthesiology and intensive care. An interview guide with open‐ended questions was used (Appendix S2). The interview guide was informed by literature on disaster preparedness and ICU resilience and refined in discussion with the research team. A pilot interview was conducted to test clarity and relevance. One pilot interview was conducted and evaluated. In total, 10 face‐to‐face interviews were conducted in a private space at the participant's workplace. One interview was conducted via the digital platform Microsoft Teams. Each interview lasted 35–45 min and was audio‐recorded and transcribed verbatim. No repeat interviews were made. Participants were pseudonymised using alphanumeric codes (e.g., A1). Transcripts were named A1, A2, etc. to denote participant number, according to the chronological order in which the interviews were made. All interviews were conducted and transcribed by the same person (PKN). All participants were given the opportunity to review their transcript; however, no one requested to review their interview. Recordings and transcripts were securely stored on encrypted servers, accessible only to the research team. Data saturation was considered reached when no new codes or themes emerged, as judged by the research team during iterative analysis.

### 2.5 Data Analysis

Each interview was first analysed in its entirety. Further analysis was done through content analysis inspired by Graneheim and Lundman [14]. In this way, the transcribed material was first divided into units of meaning and given a descriptive code. Based on patterns, similarities and differences, relevant categories and subcategories were created with the aim of the study in mind. Codes, categories and interpretations were regularly discussed and reviewed within the research team. After conducting 11 interviews, no additional information was obtained, and the research group judged that saturation of the material had been reached [6]. Therefore, no further interviews were conducted. Analysis was informed by resilience theory and adaptive governance. Resilience theory is a framework for understanding how systems respond to change, shocks and disturbances while maintaining their core functions and structures [15]. Adaptive governance addresses risk and uncertainty in a crisis by integrating institutions, networks, leadership, and structures, while promoting the equitable distribution of resources through systemic and contested processes [16]. These frameworks helped identify patterns of adaptation, leadership dynamics and emotional responses to systemic strain. Trustworthiness was addressed by applying strategies described by Graneheim and Lundman [14], including iterative team discussions for credibility, maintaining an audit trail for dependability and providing contextual details to support transferability.

### 2.6 Trustworthiness

Credibility was supported by purposive sampling across professions, iterative team‐based analysis and detailed documentation of analytic steps. Transferability was promoted through rich contextual descriptions of the ICU setting and participants. Reflexivity was maintained through ongoing discussion of researcher preconceptions and positionality within the team.

### 2.7 Ethical and Institutional Approvals

This study was approved by the Swedish Ethical Review Authority (Dnr 2020‐01572). Approval date: 24 October 2022.

## 3 Results/Findings

We organised three main categories that described the participants' experiences on different levels: ‘organisational level’, ‘team level’ and ‘individual level’ (Table 2). The number following each quote indicates the participant code.

**TABLE 2:** Main domains and themes organised in the analysis.

Domain	Themes
Organisational level	Organising ICU expansion: Navigating the new terrain of ICU expansion Organisational resilience: Navigating the psychological terrain of ICU work Strategic staffing: Orchestrating Organisational resources in ICU Directive dialogues: Steering ICU communication and governance
II Team level	Collaborative synergies: Enhancing ICU team dynamics Allied assurance: Cultivating support among ICU colleagues
III Individual level	Distress dynamics: Confronting uncertainty and moral challenges in intensive care Adaptive strategies: Shaping resilience in ICU settings Mid‐crisis recuperation: Facilitating recovery for ICU staff

*Note:* This table outlines the three main thematic categories—organisational, team and individual levels—along with their respective subcategories, derived from qualitative content analysis of interview data.

Abbreviation: ICU, intensive care unit.

### 3.1 Organisational Level

This domain includes a description of how staff perceived escalation, staffing and leadership.

#### 3.1.1 Organising ICU Expansion: Navigating the New Terrain of ICU Expansion

All participants described the need to expand into a larger space. There was simply no room for expansion in the existing ICU facilities.

Well, I don't think there were any alternatives. After all, patients were pouring in, and we had to take care of them. (A10)

Most participants stated that a plan for the expansion of the department was put in place early. The plan was implemented, and the expansion to new locations itself was straightforward, efficient and without major complications. However, the period after the expansion was characterised as more chaotic and less well‐planned. Participants described arriving at new locations and receiving little or no introduction. Storage and crash carts were not prepared; structures for meetings and reporting were not in place and guidelines did not yet exist. Several participants mentioned difficulties in keeping track of what was going on when the department was spread out over several locations. Overall, the first few weeks in the new environment were described as frustrating, stressful and time‐consuming.

I'm used to finding everything, and then suddenly I'm running around in four different storage rooms, just looking, but I can't find things. I think that was the most frustrating thing. (A3)

The first day was completely chaotic. I didn't even know where I was going, so I followed my colleagues. The first thing they said was that we had to go to the patients and replace our colleagues because no one had eaten lunch yet. We didn't even get a tour. (A2)

Participants had different opinions about the new wards. There were major advantages, such as good light, large areas and good conditions for separating infectious patients from non‐infectious patients. The open rooms with cohort care made it easier for staff to get help from each other. There were also clear disadvantages, such as a lack of electrical outlets and sinks, high noise levels in the open landscape and the risk of tripping on the temporarily drawn cable. One ICU had a preparation room where all procedures were performed before the patient was moved to their ICU bed. This workflow created efficiency and structure.

Yes, there was plenty of space with large areas so you could move around and work with the patients from different angles. Lots of light. Then, there was a lack of electrical outlets, cables crisscrossing everywhere, and ventilators that we didn't recognize. (A4)

Participants emphasised the need to control the entire transport chain when moving seriously ill patients. For example, staff often used public elevators with multiple stops in the transport flow because these elevators were used extensively.

Participants reported a lack of equipment for the ICU. Old ventilators or anaesthesia ventilators had to be used, which made work difficult and was sometimes potentially harmful to patients, as staff were unfamiliar with the settings and alarm systems of the equipment. A maximum of four different ventilators were used in the same ICU to manage patient overload. The filters for the ventilators and dialysis machines were used longer than recommended, and on several occasions, medications were labelled in foreign alphabets. Participants identified these examples as both a potential risk for complications for patients and stressful for staff. The participants strongly advocated better stockpiling of medical supplies in the event of a crisis.

The work environment was described as terrible, a disaster, confined, overworked, with long shifts in heavy protective equipment, short breaks, very care‐heavy patients and difficult to be heard in large, noisy environments. Several participants considered the early introduction of the Protective Mask 90 to be problematic. Participants described the mask as isolating, difficult to talk through, causing neck problems and frightening for the patients. On several occasions, participants removed their masks to communicate better with each other despite the risk of becoming infected themselves.

I remember it was very hard—you couldn't just go out and drink, go to the bathroom, or eat. I felt very confined. (A1)

No, the mask was terrible. No one could hear my voice. I remember writing on paper to communicate, and there were several times when I took the mask off during critical situations. (A11)

Finally, the participants expressed a desire for a structured evaluation of the different ICUs, highlighting what had been done well and what could have been done differently. They were convinced that they would face other extraordinary events in their careers and requested a well‐communicated plan for possible future situations.

#### 3.1.2 Organisational Resilience: Navigating the Psychological Terrain of ICU Work

Overall, there was strong confidence in immediate management to solve the problems that arose and to prioritise their staff. Many participants highlighted management's physical presence as incredibly supportive and essential to staff trust and compliance.

I have actually learned how important it is to have the support and transparency of management to be able to do something like this at all. (A5)

On the other hand, there was mistrust of the personal protective equipment (PPE) guidelines. Participants felt that there was insufficient PPE in place and that the guidelines were changed quickly depending on availability rather than to protect staff. This made participants feel unsafe because they did not trust the PPE that was available.

We saw pictures from China and Italy. There, they worked dressed as astronauts. And here, at the beginning, we would only have taped gloves. (A7)

Participants indicated that support activities were established during the early phase, mainly in the form of group counselling. There was a desire for more individualised forms of support and training in crisis management among staff accustomed to working in disaster situations.

#### 3.1.3 Strategic Staffing: Orchestrating Organisational Resources in the ICU

The participants were generally positive about the provision of new staff. This relieved the ICU staff of many tasks, allowing them to concentrate on ICU‐specific tasks and supervision.

Participants felt it was necessary to activate the emergency contract, even if this meant longer and harder working hours. For those who did not activate the emergency contract, the workload was described as overwhelming and almost impossible, which quickly changed once the contract was activated. In this context, the importance of the voluntary nature of the emergency contract was also emphasised as essential to maintaining staff morale and trust in management.

The disadvantage highlighted by the voluntary nature of the contract was the high staff turnover, with staff from other clinics rotating in for short periods, which, while helpful, also became a major challenge for ICU staff who had to constantly be responsible for new colleagues who had little or no introduction. There was a desire for greater continuity in the provision of staff for similar needs in the future.

As a sole physician, being responsible for all these scattered patients was almost impossible. But when we switched to the emergency contract, we had several teams there at night. It was a huge relief. (A8)

Because of the volunteer approach, the anesthesia staff wanted to rotate in for two weeks, and then new staff would replace them. And we felt that now they were here, and they were on board. Why can't they just stay here with us? Of course, it was an advantage, because then the staff could last longer, but it was also a challenge for ICU staff, who had to onboard and instruct many more people. (A7)

#### 3.1.4 Directive Dialogues: Steering ICU Communication and Governance

Participants described how the leadership was able to act early in the escalation by increasing the number of beds in the ICU, recruiting colleagues and purchasing equipment as quickly as possible. Participants also commented that decision‐making became very hierarchical as hospitals went into reinforcement mode. Some participants likened this to a war situation. The organisation also became very solution‐oriented and dynamic. It was emphasised that this was necessary to create an efficient organisation and to allow staff to focus entirely on clinical work.

So now we have superiors, and we as subordinates are supposed to obey orders. And this should be as effective as possible. And I don't remember anyone questioning the decisions. (A11)

Other participants mentioned being part of the management team one day, gaining full insight into decision‐making and then being completely excluded the next day as leadership became more streamlined. This sparked numerous thoughts and ideas on how the organisation could be optimised. There was a desire for new ways of working to make better use of existing expertise for future exceptional events.

Participants reported considerable frustration when hospital management did not escalate to disaster mode. The National Board of Health and Welfare in Sweden defines ‘disaster mode’ as the highest level of preparedness a Swedish hospital can reach. This means that all necessary medical functions are activated to treat a large number of casualties, as well as an increase in personnel and equipment. It also posed a significant ethical challenge to be expected to provide adequate medical quality when access to both equipment and expertise was compromised.

But if those in power say that the quality of health care is maintained when it is not, then the people whose heads are rolling are the ones who work on the ground. That's the cynical reality, and that's really the betrayal of the staff in the region. (A8)

The information provided by management to staff was generally described as transparent, structured and adequate. In situations where the plans were not properly communicated, there was anxiety, uncertainty and sometimes, false rumours. For instance, the communication to ICU staff and anaesthesia staff could vary, because they had different managers. It was emphasised that it is very important to have transparent and coordinated communication with all collaborating clinics to create security, understanding and trust in the staff group.

Finally, the participants suggested the need for more recurring scientific forums with information about the latest knowledge. Knowledge transfer was described as low, especially between different professions.

### 3.2 Team Level

This domain includes collaboration within the team, within the staff group and between professions, as well as experiences of collegial support.

#### 3.2.1 Collaborative Synergies: Enhancing ICU Team Dynamics

Overall, participants described good collaboration within the hospital under the given conditions. The organisations had to quickly develop very clear routines and procedures for how to communicate and report, as well as who had to do what. Over time, the ICU staff became the leaders, taking on the roles of supervising, delegating, educating and being specifically responsible for the ICU. This clear working arrangement took time to establish, but once in place, it created a greater sense of security, dynamism and creativity in the teams working together.

Several participants emphasised the rich flow of creativity at a time when much was unknown about the disease. The focus was on solving emergency situations and saving as many patients as possible. In contrast to the organisational hierarchy that emerged, many participants described how hierarchy and prestige within the teams disappeared and more colleagues stepped up to show their strengths.

Then, you just have to see what skills people have when you need them. Then researchers and logisticians show up, as well as medical professionals, creative minds, and ethical concerns. Suddenly, we have what we need to take care of this. (A6)

Within the Stockholm region, however, collaboration was characterised as more cumbersome. Participants felt that the different hospitals prioritised their own units, and it became complicated to transfer ICU patients to other hospitals. The participants expressed a desire for better and more independent regional and, to some extent, national governance.

#### 3.2.2 Allied Assurance: Cultivating Support Among ICU Colleagues

Participants described a calmness among the staff group to face this together, due in large part to a well‐functioning clinic, good colleagues and high trust in immediate management. Good support from their own colleagues and other professionals was highlighted as an important factor in coping with such a difficult challenge. Colleagues became the support that family, friends, counsellors and the rest of the world could not provide. This situation was described as exciting and historic, which fostered a sense of community and closeness in the group. Conversations became deeper, and colleagues allowed themselves to show their vulnerability. Participants reported growing closer to their colleagues, both in the ICU and in the hospital.

We became closer because we were going through something like this, historically, traumatically, and hugely together. (A1)

At the same time, participants spoke of loneliness. The transfer of knowledge deteriorated, the team around the patient weakened, and instead, staff became more isolated in their own profession. Competence was described as scarce, so there was no time or space for discussion. This made it more difficult to make balanced medical and ethical decisions by consensus. This could manifest itself, for example, in patients being cared for too long without a clear plan or in well‐formulated treatment decisions being torn up.

Sure, we were well staffed, but at the same time, we were alone in terms of expertise. (A2)

Another effect of loneliness was that ICU nurses felt particularly marginalised. They had to carry a heavy burden of responsibility in bedside care, as they were responsible for many new colleagues. At the same time, they did not receive the same support from the medical team as they were used to, which led to isolation and exclusion. It was emphasised that better interprofessional collaboration would have been desirable.

### 3.3 Individual Level

This domain summarises the participants' experiences of working in a completely new situation, the moral stress that arose, the ability to adapt to the new conditions and the possibility of recovery.

#### 3.3.1 Distress Dynamics: Confronting Uncertainty and Moral Challenges in the ICU

During this period, the participants reported considerable ethical and moral distress at work. There was a sense of powerlessness in the face of the large influx of patients who were so seriously ill that it was difficult to find emergency equipment when needed, and at the same time, the staff could not provide the advanced care that they normally did. Patients' conditions could quickly deteriorate to life‐threatening levels, and in the early stages, no one really understood why, which caused a great deal of anxiety and frustration among the staff. Participants expressed a deep‐seated apprehension about mistreating patients, recounting instances where patients were admitted to the ICU for completely hopeless treatment or were treated for far too long. There was also a fear of contracting the virus, becoming ill themselves, or infecting their families.

So, it wasn't a mixture of fear and joy, but it was more fear than joy. (A10)

I felt like I was in a war, and I feel like I'm the most useless war nurse ever. (A2)

#### 3.3.2 Adaptive Strategies: Shaping Resilience in ICU Settings

The participants talked about their different ways of adapting to the extraordinary situation that had arisen. Some described themselves as pragmatic and said that they could only do their best under the circumstances. It was like a state of war where the most important things had to be prioritised. It was a difficult but exciting time, with many creative solutions.

Another participant reported a considerable amount of frustration that patients were not receiving adequate care and that there was a lack of time and skill for each patient. The participant described a need for control and difficulty in delegating, which led to significant moral distress.

But then there was the virus. That was impossible. No, I mean, we weren't good. Yes, we sucked. We should not be written about as heroes. We were confused little caregivers who went around and tried to put out fires and could have done it so much better, I think. (A11)

If it's going to be like this, then I picture the patients as logs in the beds or something, because if you are going to be able to cope with this and that it would get this hard, then you have to defend yourself somehow. (A4)

#### 3.3.3 Mid‐Crisis Recuperation: Facilitating Recovery for ICU Staff

Staff recovery was unanimously described as non‐existent. Participants emphasised that working long shifts consecutively, with only occasional days off in between, provided no opportunity for rest. Participants described how their energy was spent working, eating and sleeping. Most of their free time was spent resting. Some participants quickly realised that they needed to work regular hours to sustain themselves over time.

There was no recovery. When I had worked seven weeks on the emergency contract, I was so tired that I couldn't even sit up, so instead, I was just lying on the couch resting. (A2)

In one hospital, management decided early in the first wave to move staff from the regular 12‐h shifts stipulated in the emergency contract to 8‐h shifts. The goal was to reduce the burden of long working hours and allow for more overlap between colleagues. These overlaps allowed team members to cover for each other during breaks and recovery periods. Several participants stated that this shift adjustment had a positive effect on their recovery process.

## 4 Discussion

In this study we show that experiences of ICU staff in Sweden during a state of near‐disaster included challenges related to limited resources, adapting to new procedures, leadership and communication, collaboration and collegial support and moral distress. Our findings illustrate resilience as the capacity of ICU staff to adapt workflows, maintain cohesion and preserve core functions despite systemic strain, aligning with established resilience frameworks.

### 4.1 Organisational Level

ICUs require substantial resources, and in disasters the model of care may shift towards crisis standards of care, reallocating resources to manage surges [1, 17, 18]. Planning should therefore clarify the thresholds for implementing and ending crisis standards, alongside governance changes needed to support staff. Previous literature highlights gaps in governance during such transitions and the strain this imposes on healthcare workers [1]. During the first COVID‐19 wave, Swedish disaster mode—the highest level of preparedness—was not activated, as the pandemic was framed as a prolonged emergency. This decision created ethical and operational challenges for frontline staff, underscoring the need for clearer criteria and transparent disaster activation in future crises.

Participants reported that ICU expansion proceeded smoothly at first but was followed by challenges, with inadequate preparation, lack of equipment and unsafe reliance on outdated ventilators. These findings align with prior studies advocating for robust stockpiles of medical and protective equipment [19]. Leadership presence, prioritisation of staff well‐being and clear communication were emphasised as critical supports, consistent with earlier research on effective crisis leadership [20, 21]. However, mistrust grew around shifting PPE guidelines, perceived as driven by availability rather than safety. Transparent, consistent communication was highlighted as central for trust and cohesion, a point supported by previous studies [22].

Governance challenges were evident. Participants described frustration when hospitals refrained from declaring disaster mode, leaving them expected to deliver standard care under extraordinary circumstances. This strained both morale and ethics. The results point to the importance of hybrid governance: blending hierarchical command with frontline expertise to support adaptability. Such structures may ensure that staff perspectives are considered while maintaining organisational efficiency [23].

### 4.2 Team Level

Team dynamics played a decisive role in resilience. Over time, ICU staff developed clear routines and communication channels that improved coordination. Interestingly, peer hierarchy diminished while organisational hierarchy intensified: frontline collaboration became more egalitarian, enabling creativity and problem‐solving, while hospital‐level governance became top‐down. Balancing these dimensions—integrating command structures with frontline flexibility—appears critical to crisis management [23].

Collegial support was consistently described as a lifeline. Shared purpose created strong bonds and morale, aligning with studies showing enhanced collaboration during crises [24, 25]. Yet over time, interprofessional teams fractured. Knowledge transfer deteriorated, and ICU nurses in particular reported feeling isolated when tasked with supervising untrained staff without adequate medical support. Improved interprofessional collaboration and structured opportunities for debriefing could mitigate these risks.

The influx of non‐ICU‐trained staff was a double‐edged sword: essential for capacity but requiring supervision that burdened experienced ICU staff. Constant rotation of redeployed staff undermined continuity and team cohesion. Previous research underscores the importance of stable, cohesive teams in high‐stress environments [26]. Similar dynamics were seen in Ukraine, where specialists outside intensive care had to adapt rapidly to critical care roles [3]. Strategies for recruiting staff for longer terms, coupled with rapid onboarding and simulation‐based training, could strengthen future responses.

ICU expansion into new spaces created additional barriers to teamwork. Staff had to navigate unfamiliar environments with limited space for recuperation or debriefing. These conditions exacerbated miscommunication and inefficiency. Evidence from UK and Brazilian studies corroborates the importance of teamwork and leadership as key resilience factors in pandemic ICUs [27, 28, 29]. Our findings suggest that future disaster planning should prioritise designated collaboration spaces and structured routines for interprofessional communication.

### 4.3 Individual Level

At the individual level, participants reported profound adaptation challenges and moral distress. Staff faced powerlessness when unable to provide optimal care due to resource shortages and systemic constraints. This aligns with Jameton's original concept of moral distress—knowing the right course of action but being unable to pursue it due to institutional barriers [30, 31]. Similar experiences have been documented across ICU staff globally [26, 32, 33]. The moral burden was particularly heavy when governance failed to activate disaster protocols, leaving staff to bear the responsibility for substandard care. Literature confirms that governance gaps during crisis standards can intensify moral distress and negatively affect healthcare practitioners [1, 34].

The absence of early mental health resources compounded these burdens. Participants called for tailored psychological support and crisis management training, echoing recommendations from other studies [25, 35]. Organisational preparedness should include rapidly deployable mental health programmes during crises.

Despite exhaustion, participants described a sense of commitment and meaningfulness, consistent with prior research [25, 35]. Coping strategies varied widely: some staff found motivation and creativity in the crisis, while others experienced inadequacy and distress. Personality, coping style and resilience‐building interventions play crucial roles in managing stress under pressure [36]. Interventions to foster resilience and stress management should be continuous and not limited to acute crises.

Recovery opportunities were limited. Prolonged long shifts without rest led to exhaustion, consistent with evidence linking inadequate recovery to burnout and reduced patient safety [37]. Some units mitigated this by moving to shorter shifts with overlap, which improved recuperation and teamwork. Policies ensuring recovery time, even in crises, should be integral to preparedness planning.

Finally, participants stressed that the failure to activate disaster protocols deprived them of structured support, flexible staffing and formal debriefing. Their repeated description of a ‘war zone’ reflects the chronic nature of the COVID‐19 surge. Unlike acute disasters, the pandemic created sustained disaster‐level strain, supporting its framing as a chronic major incident. Recognising such prolonged crises as disasters is essential for mobilising systemic support and preventing staff harm. Future research should explore how to adapt disaster protocols not only for sudden surges but also for extended crises.

## 5 Limitations

This study has some limitations. First, interviews were conducted 2–3 years after the near‐disaster, which may have introduced recall bias by dampening emotional detail or idealising leadership. Yet, the time gap may also be a strength, as participants had processed the trauma and provided more reflective accounts [38]. Second, the small sample may underrepresent certain groups, such as night‐shift staff, limiting generalisability. However, qualitative research emphasises transferability rather than population‐level generalisation, leaving it to the reader to interpret findings in context [14]. Finally, we focused on experiences of escalating care during the near‐disaster, not the pandemic as a whole. Participants sometimes struggled to separate these, making extrapolation to future crises more complex in a low‐disaster risk setting like Sweden.

## 6 Recommendations or Implications for Practice and/or Further Research

Hospitals should implement rapid onboarding for non‐ICU staff, activate tailored mental health support early and establish clear criteria for disaster protocol activation. Leadership training should include crisis communication and moral distress management. Future research should explore how hybrid governance models function in practice and assess long‐term psychological impacts on ICU staff after near‐disaster events.

## 7 Conclusion

Across organisational, team and individual levels, ICU staff demonstrated resilience through adaptation, creativity and commitment, despite governance gaps and systemic strain. Organisational resilience requires clear criteria for activating disaster protocols, transparent communication and hybrid governance structures that balance hierarchical command with frontline expertise. At the team level, continuity, interprofessional collaboration and structured knowledge transfer are vital. Individually, supporting staff through resilience training, adequate rest and rapid psychological support is essential to mitigate long‐term harm. Lessons from this near‐disaster extend beyond COVID‐19, offering guidance for building more robust and humane disaster preparedness systems in Sweden and comparable contexts.

## Author Contributions

**Per Kolton Nyberg:** data curation, formal analysis, writing – original draft, writing – review and editing. **Mattias Günther:** conceptualization, supervision, writing – review and editing. **Ami Fagerdahl:** conceptualization, methodology, formal analysis, writing – review and editing. **Andreas Älgå:** conceptualization, methodology, supervision, writing – review and editing.

## Ethics Statement

This study was approved by the Swedish Ethical Review Authority (Dnr 2020‐01572). Approval date: 24 October 2022. Written consent was obtained from all participants prior to the study.

## Consent

The authors have nothing to report.

## Conflicts of Interest

The authors declare no conflicts of interest.

## Supporting information


**Appendix S1:** COREQ (Consolidated criteria for reporting qualitative research) checklist.

**Appendix S2:** Interview guide.

## Data Availability

The data that support the findings of this study are available on request from the corresponding author. The data are not publicly available due to privacy or ethical restrictions.
