# Supplementary material for: Beyond Capacity: Resilience of Intensive Care Staff During the COVID‐19‐Related Near‐Disaster in Sweden
Source: Nurs Crit Care. 2025 Nov 17;30(6):e70251. doi: 10.1111/nicc.70251 (PMC12621160; doi:10.1111/nicc.70251)
Supplement: Supplementary file 2 — Appendix S2: Interview guide. [file NICC-30-0-s002.docx]

Appendix - Interview Guide

1. Now, let’s go back to the spring of 2020. You are about to admit your first COVID-19 patient to the ICU.
   1. Tell us, how did it feel to get the news that the capacity of the ICU had to be increased so quickly?
   2. Was it a reasonable decision to escalate as it was?
   3. Information?
   4. How did you experience the escalation itself?
      1. Collaboration, on the floor, with managers?
      2. Problems arose?
2. In just a few weeks, your ICU escalated from xx ICU beds to a total of xx ICU beds. How do you remember that time?
   1. Biggest challenges?
   2. Unknown situations?
      1. Safety?
      2. Control?
   3. Sense of loneliness (competence, physical)?
   4. Patient safety?
   5. Equipment, something missing?
   6. New colleagues?
   7. Mood?
3. How did you find the work environment in general?
   1. Emergency contract?
   2. Staffing?
   3. Stress level?
   4. Recovery?
   5. Counseling contact, counseling at home?
4. How did communication with managers work?
5. Do you think anything could have been done differently?
   1. Patient care?
   2. Information?
   3. Escalation?
   4. Scheduling?
6. What is the most important message you took away from the first wave of the pandemic?

# Supporting Information

# Supinfo1
